# Increasing bystander CPR: potential of a one question telecommunicator identification algorithm

**DOI:** 10.1186/s13049-015-0115-1

**Published:** 2015-05-13

**Authors:** Ross Orpet, Randi Riesenberg, Jenny Shin, Cleo Subido, Eddie Markul, Thomas Rea

**Affiliations:** From the University of Washington, Seattle, USA; Emergency Medical Services Division of Public Health – Seattle & King County, ᅟ, ᅟ; University of Illinois at Chicago, Chicago, USA

**Keywords:** Cardiac arrest, Cardiopulmonary Resuscitation (CPR), Diagnosis, Dispatcher

## Abstract

**Objectives:**

Telecommunicators use a two-question algorithm to identify cardiac arrest: *Is the individual conscious? Is the individual breathing normally?* Although this approach increases arrest identification and consequently bystander CPR, the strategy does not identify all arrests and requires time to complete. We evaluated the implications of a one-question strategy that inquired only about consciousness.

**Methods:**

We undertook a 3-month observational study of consecutive cases identified as unconscious by the telecommunicator prior to EMS arrival who were not receiving bystander CPR. We evaluated the extent that a one-question strategy could increase arrest identification and reduce the identification interval; and the trade-off whereby additional persons without arrest could potentially receive CPR.

**Results:**

Among 679 eligible cases, 20% (n = 135) were in arrest and 80% (n = 544) were not in arrest. The two-question algorithm identified 90% (121/135) as true arrest. Of the 135 in arrest, 70% (n = 95) received compressions. The median interval from call to arrest identification was 72 seconds, with a median of 14 seconds for the breathing normally question. Using the two-question algorithm, telecommunicators incorrectly classified 30% (n = 164/544) of non-arrests as arrest. Bystanders proceeded to compressions in 16% (n = 85/544) of persons not in arrest. A one-question approach that inquired only about consciousness could potentially increase the arrest identification by 10% (14/135) and reduce the interval to compressions by a median of 14 seconds; however the strategy would potentially triple the number of non-arrest cases (544 versus 164) eligible for CPR instructions.

**Conclusion:**

A single-question arrest identification algorithm may not achieve a favorable balance of risk and benefit.

## Background

Cardiac arrest is an unexpected event in which a person’s heart suddenly stops beating effectively. Out-of-hospital cardiac arrest accounts for hundreds of thousands of deaths each year in the US and around the world [1]. Survival can be improved if cardiopulmonary resuscitation (CPR) is provided to the victim soon after collapse [2]. Training of the telecommunicator to quickly identify potential cardiac arrest victims and provide CPR instructions over the phone can increase the proportion who receive early bystander CPR and is associated with an increase in cardiac arrest survival [3]. However, cardiac arrest can sometimes be difficult to distinguish from other conditions that do not require CPR, such as seizure or hypoglycemia reaction. Currently, in most protocols the telecommunicator prioritizes a sequential 2-question algorithm in an attempt to identify potential cardiac arrest patients:

### Is the individual conscious? Is the individual breathing normally?

If the answer to both of these questions is “no”, then the telecommunicator engages the caller to provide CPR.

There are often challenges to comprehensive and timely arrest identification even with consistent use of the 2-question algorithm. Prior investigation indicates telecommunicators fail to recognize cardiac arrest in a quarter to half of cases, most often because the caller reports potential signs of life – typically patient breathing [4,5]. Moreover, the determination of breathing status can require a minute or more and thus delay the onset of CPR [5].

One strategy to overcome these challenges to early arrest identification and early bystander CPR is to eliminate the question about normal breathing and simply consider CPR for any patient that is reported as unconscious. However, there is no information regarding the balance of benefit and risk involving a single question strategy for identification. The strategy may enable more comprehensive and timely bystander CPR but presumably would initiate CPR in a larger group without arrest. Although telecommunicator CPR is generally safe, risk of injury is not zero [6].

We undertook an observational study to evaluate the implications of using a one-question algorithm for identifying arrest patients. Specifically we wanted to evaluate the extent that a one-question strategy that inquired about consciousness would increase arrest identification and reduce the interval from call to arrest identification; and the trade-off whereby additional persons without arrest would potentially receive CPR unnecessarily.

## Methods

### Study design, population, and setting

The investigation is a retrospective cohort study of consecutive 9-1-1 emergency calls where the patient was determined to be unconscious between February 1, 2013 and April 30, 2013 at two telecommunication centers in a large metropolitan county. The study was approved by the investigational review board of the University of Washington and Public Health – Seattle & King County. For each medical emergency call, telecommunicators electronically enter an initial dispatch code that specifies whether the patient was conscious. We excluded those cases that were determined by emergency medical services (EMS) to have irreversible death upon their evaluation, cases that were transferred from outside dispatch centers or occurred after EMS arrival, cases where CPR was ongoing at the time of the telecommunicator assessment, and cases where the recording could not be reviewed because of poor quality audio or other technical reasons.

The two centers serve a population of about 1.2 million persons who reside in urban, suburban, and rural areas of King County, covering approximately 2000 square miles. The telecommunication centers handle police, fire, and medical emergency calls. Telecommunicators undergo 32 hours of initial emergency medical telecommunicator training, including 6 hours dedicated to the recognition of cardiac arrest and the delivery of CPR instructions. The telecommunicators complete 8 hours of continuing education annually and participate in a quality assurance program that provides feedback about telecommunicator CPR performance.

### Telecommunicator CPR

Telecommunicators in the study community use a standard Criteria-Based Dispatch protocol that prioritizes the identification of cardiac arrest by using a two question algorithm. The telecommunicator first asks if the patient is conscious. If the answer is no, then the telecommunicator asks if the patient is breathing normally. If the caller answers no, then the telecommunicator engages the caller to provide CPR.

### Data collection and definitions

The EMS system maintains a registry of each EMS-treated out-of-hospital cardiac arrest by reviewing telecommunicator audio recordings, EMS written reports, electronic defibrillator downloads, and hospital records. A cardiac arrest is defined as a pulseless patient requiring EMS CPR or a patient who received a public access defibrillator shock prior to EMS arrival. The information is organized according to the Utstein template and data definitions [7]. For calls that are not cardiac arrest, the EMS maintains information about patient demographic characteristics, vital signs, prehospital diagnostic codes, EMS care, and prehospital outcome.

For the purposes of the current investigation, we reviewed the audio recordings using a uniform abstraction form to determine when the patient was determined to be unconscious, when the telecommunicator inquired about normal breathing, when the patient was determined to be breathing normally or not breathing normally, when CPR instructions were offered (if appropriate), and when chest compressions were initiated (if appropriate). One investigator (RO) reviewed all cases. A second investigator (RR) independently reviewed 20 cases. The inter-rater agreement was 100% regarding the qualitative data elements i.e. did the telecommunicator inquire about consciousness, inquire about normal breathing, and offer CPR instructions. Agreement with regard to timing for these data elements was within 5 seconds in 90% of cases.

### Statistical analysis

We used descriptive statistics to determine what proportion of study cases identified with the two-question and potential single-question algorithm had true cardiac arrest versus another condition as well as the timing of telecommunicator and caller actions. We stratified the evaluations of timing according to true arrest and non-arrest status.

## Results

**Figure 1:**
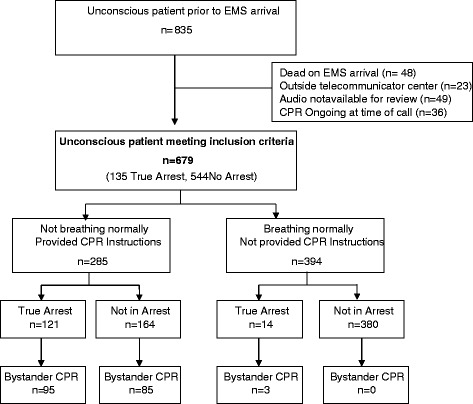
Cardiac arrest according to consciousness and breathing status. Interquartile range is abbreviated IQR.

A total of 835 calls occurred before EMS arrival and were coded as unconscious. Of these 835, 48 were determined to have irreversible death upon initial evaluation by EMS, 23 had been processed already and were relayed from outside centers, 49 audio recordings could not be located for review or were technically inadequate for abstraction, and 36 had CPR ongoing at the time of initial telecommunicator inquiry that negated the need for the two question algorithm (Figure 1). In addition, there were another 37 cardiac arrest before EMS arrival who were initially coded by the dispatcher as conscious at the outset of the call. Thus during the study period there were a total of 208 cardiac arrests prior to EMS arrival.

Among 679 cases deemed not conscious by the telecommunicator, 135 (20%) were in arrest and 544 (80%) were not in arrest. Of the 679, 285 were identified as not breathing normally by the telecommunicator using the two question approach, so considered potential cardiac arrest and offered CPR instructions, while 394 were assessed to be breathing normally so not offered instruction (Figure 1). Among those 285 who were offered instructions, 121 were in true arrest and 164 were not in arrest. Of the 285 offered CPR instructions, 180 actually received chest compressions; 95 who were actually in arrest and 85 who were not in arrest.

Using a single question algorithm, all 679 cases would have been eligible for telecommunicator CPR and theoretically offered CPR instructions (Figure 1). Of these 679, a total of 135 cases (20%) actually experienced cardiac arrest, 14 of whom were assessed by telecommunicators as breathing normally. Thus a single question algorithm that inquired only about consciousness could potentially increase the identification of true cardiac arrests by 10% (14/[121 + 14]), but potentially would have provided CPR instructions to an additional 380 (679 – [285 + 14]) who were not in arrest. Thus, the one-question algorithm would potentially triple the frequency of offering CPR instructions to patients not in arrest (380 + 164 compared to 164).

**Figure 2:**
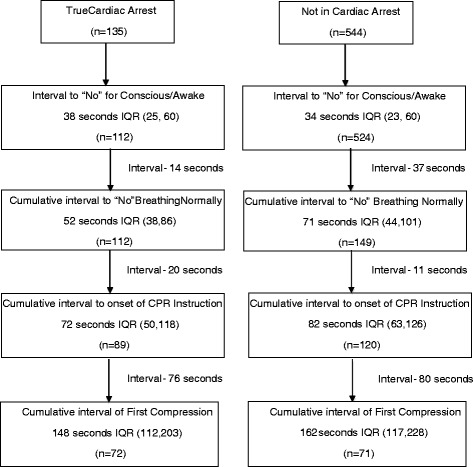
Median intervals from call receipt to chest compressions according to arrest status. Interquartile range is abbreviated IQR.

Figure 2 presents the results for the time intervals from call receipt through delivery of chest compressions stratified by arrest status. For patients in true cardiac arrest, the telecommunicator determined that the patient was not conscious in a median of 38 seconds after call receipt. The telecommunicator determined that the patient was not breathing normally in a median of 52 seconds after call receipt, or a median of 14 additional seconds after the telecommunicator determined the patient was not conscious. Chest compressions were actually begun in a median of 148 seconds after call receipt. A portion without cardiac arrest also proceeded to chest compressions using the two question algorithm in a median of 162 seconds after call receipt. The most common EMS-based diagnoses of patients without arrest were overdose (20%), syncope (10%), seizure (8%), other neurologic emergency (7%), and hypoglycemia (6%).

## Discussion

In this cohort of consecutive cases determined to be unconscious by telecommunicators, 20% (135/679) were in true cardiac arrest and 80% (544/679) were not in arrest. In this unconscious cohort, telecommunicators using a two-question algorithm identified 90% (121/135) of true arrests and coached bystander compressions in 70% (95/135) of true arrests initially identified as unconsciousness and not already receiving CPR. Using this two-question algorithm, telecommunicators incorrectly classified 30% (n = 164/544) of unconscious persons not in arrest as a cardiac arrest patient and bystanders proceeded to start compressions in 16% (n = 85/544) of this non-arrest group. A one-question approach that inquired only about consciousness could potentially increase the sensitivity of arrest identification by about 10% (14/135) and reduce the interval to compressions for those with true arrest by approximately 15 seconds; however the strategy would potentially triple the number of non-arrest cases who could receive CPR instructions.

The purpose of this investigation was to provide a framework for how a one question algorithm might impact early bystander CPR for those with and without cardiac arrest compared to the standard two question format. The two-question algorithm identified and enabled bystander CPR in most eligible cardiac arrests with the first compression occurring in about 2.5 minutes after call receipt. About a third of patients who received chest compressions were not in true arrest. These results are comparable to prior results and provide assurance that the performance of the 2-question algorithm is reproducible and that performance standards are relevant [4,8].

In contrast, a single question algorithm that inquired only about consciousness increased timely identification of true arrest by about 10% but with substantial “false positive” identification, which would likely lead to chest compressions in substantially more persons not in arrest. Bystander CPR rarely causes serious injury [6]. However, the current study suggests that a single-question algorithm, robustly implemented as part of guidelines and incorporated into dispatch protocols, could translate to a substantial increase in false-positive bystander CPR. When extrapolated on an international scale, such a change in identification - even if incidence of serious injury is rare - could produce excess meaningful and measurable adverse outcomes in the non-arrest group.

The current study has limitations. The study occurred in a mature EMS system where telecommunicators are trained in CPR instruction and participate in a quality assurance program to improve care for cardiac arrest. These characteristics helped facilitate the current study, but may limit the generalizability of the findings. The investigation was retrospective and in fact did not actually implement a single question algorithm. As a consequence we can only estimate the numbers that would be identified as arrest and potentially proceed to actual bystander CPR using this strategy. The triaging algorithm produced small numbers for some of the cells i.e. “breathing normally” and in true arrest. This small count is in part due to the performance characteristics of the two-question algorithm but the modest numbers also can limit the confidence to make inference. Although we could assess the qualitative status of CPR instruction and bystander CPR, we could not ascertain definite time points for all cases in evaluating the time course of arrest identification and provision of CPR instruction. These cases with missing time information may have been different from those with complete time information.

## Conclusion

Early CPR is a fundamental link in the chain of survival. An effective program of telecommunicator CPR is an important and effective strategy to increase bystander CPR and improve community resuscitation outcomes. Although a one-question algorithm would potentially identify additional cardiac arrests and reduce the overall time interval from call receipt to the provision of the compressions, the strategy would substantially increase the number of non-arrest patients who could receive CPR instructions. Resuscitation stakeholders need to balance benefit and risk as they consider innovative approaches to increase effective and timely CPR. A single question arrest identification algorithm may not achieve a favorable balance of risk and benefit in many communities.
